# Association Between Lifestyle Habits and the Prevalence of Abdominal Obesity After the Great East Japan Earthquake: The Fukushima Health Management Survey

**DOI:** 10.2188/jea.JE20200597

**Published:** 2022-11-05

**Authors:** Mayu Yasuda Uemura, Tetsuya Ohira, Seiji Yasumura, Akira Sakai, Atsushi Takahashi, Mitsuaki Hosoya, Masanori Nagao, Hironori Nakano, Hitoshi Ohto, Kenji Kamiya

**Affiliations:** 1 Radiation Medical Science Center for the Fukushima Health Management Survey, Fukushima Medical University, Fukushima, Japan; 2 Department of Epidemiology, Fukushima Medical University School of Medicine, Fukushima, Japan; 3 Department of Public Health and Health Systems, Nagoya University Graduate School of Medicine, Nagoya, Japan; 4 Department of Public Health, Fukushima Medical University School of Medicine, Fukushima, Japan; 5 Department of Radiation Life Sciences, Fukushima Medical University School of Medicine, Fukushima, Japan; 6 Department of Gastroenterology, Fukushima Medical University School of Medicine, Fukushima, Japan; 7 Department of Pediatrics, Fukushima Medical University School of Medicine, Fukushima, Japan; 8 Research Institute for Radiation Biology and Medicine, Hiroshima University, Hiroshima, Japan

**Keywords:** The Great East Japan Earthquake, lifestyle, abdominal obesity, health check-ups, smoking cessation

## Abstract

**Background:**

The proportion of overweight individuals living in the evacuation zone of Fukushima increased after the Great East Japan Earthquake. However, the change in the prevalence of abdominal obesity has not been reported. Lifestyle habits and changes in these habits after the disaster might have affected the onset of abdominal obesity; however, the association between the two is unclear.

**Methods:**

This study evaluated 19,673 Japanese participants of the Fukushima Health Management Survey. We used data from general health check-ups conducted in 13 municipalities between 2008 and 2010. Follow-up examinations were performed from June 2011 to March 2013. Changes in the proportion of individuals with abdominal obesity before and after the disaster were compared. Then, lifestyle habits affecting these changes were assessed.

**Results:**

We found that 34.2% and 36.6% of participants (*P* < 0.001), both evacuees (37.0% and 42.1% [*P* < 0.001]) and non-evacuees (32.8% and 34.0% [*P* < 0.001]), had abdominal obesity before and after the disaster, respectively. Abdominal obesity was positively associated with smoking cessation, snacking after dinner, and non-breakfast skipping after the disaster and alcohol drinking before and after the disaster (all *P* < 0.05). Smoking cessation was positively associated with abdominal obesity in both evacuees and non-evacuees and in both men and women (all *P* < 0.01).

**Conclusion:**

The prevalence of abdominal obesity increased among residents in the area affected by nuclear disaster. It might be associated with not only lifestyle habits before the disaster but also changes in these habits after the disaster, especially smoking cessation.

## INTRODUCTION

The Great East Japan Earthquake, with a magnitude of 9.0, occurred on March 11, 2011 in the Pacific coast of the northern area of Japan. The earthquake and the subsequent gigantic tsunami caused a serious accident at the Fukushima Daiichi Nuclear Power Plant, and more than 160,000 residents in the Fukushima Prefecture were forcefully evacuated, including 20,000 who voluntarily evacuated. The Fukushima Health Management Survey (FHMS) was performed to immediately monitor the long-term health of residents after the disaster.^1^

The FHMS showed that the proportion of overweight individuals increased among residents in the evacuation zone of Fukushima Prefecture after the disaster, and these changes were observed particularly among evacuees.^2^ Furthermore, it has been reported that evacuees and men had greater body mass index (BMI) increase and higher systolic and diastolic blood pressure than non-evacuees and women. Moreover, evacuees had a higher prevalence of diabetes mellitus after the disaster than non-evacuees.^3^^,^^4^

Abdominal obesity, similar to hypertension, hyperglycaemia, and hyperlipidaemia, is a major component of metabolic syndrome.^5^^,^^6^ Metabolic syndrome is associated with a greater risk of developing cardiovascular disease and type 2 diabetes.^7^ However, changes in the proportion of individuals with abdominal obesity after the disaster is still not elucidated.

Abdominal obesity was found to be associated with several lifestyle habits, including alcohol drinking, diet, and physical activity.^8^^–^^12^ Moreover, after the disaster, several evacuees in the Fukushima Prefecture were forced to change their lifestyle habits, such as diet, physical activity, and smoking status, due to major changes in living condition and jobs.^13^^–^^15^ These changes in lifestyle habits differ in the presence or absence of evacuation and by sex. Furthermore, the presence or absence of evacuation and sex differences are also recognised in the onset of lifestyle-related diseases.^4^

However, whether the prevalence of abdominal obesity changed after the disaster remains unclear. Furthermore, the association between lifestyle habits and incidence of abdominal obesity after the disaster has not been fully elucidated. Hence, the current study aimed to identify lifestyle habits associated with changes in the proportion of individuals with abdominal obesity living in the Fukushima Prefecture in Japan after the Great East Japan Earthquake and evaluate the difference in the presence or absence of evacuation and sex.

## METHODS

### Study population

This study included Japanese men and women living in municipalities near the Fukushima Daiichi Nuclear Power Plant in Fukushima Prefecture, which include Tamura City, Minamisoma City, Kawamata-machi, Hirono-machi, Naraha-machi, Tomioka-machi, Kawauchi-mura, Okuma-machi, Futaba-machi, Namie-machi, Katsurao-mura, Iitate-mura and Date City. All residents of Hirono-machi, Naraha-machi, Tomioka-machi, Kawauchi-mura, Okuma-machi, Futaba-machi, Namie-machi, Katsurao-mura and Iitate-mura and some residents of Tamura City, Minami-Soma City, Kawamata-machi, and Date City were forced to evacuate from their homes as the local radiation levels increased after the Fukushima Daiichi Nuclear Power Plant accident. In these municipalities, residents aged 40–74 years and elderly individuals aged ≥75 years (the target population for health check-ups comprised 18,745 men and 22,888 women, with a mean age of 67 years) underwent annual health check-ups, which are covered by the National Health Care Insurance, from June 2008 through March 2010. Follow-up health check-ups, which are part of a comprehensive assessment in the FHMS, were conducted from June 2011 through March 2013. The detailed methods of the comprehensive health check-ups have been published previously, and the follow-up survey was conducted countrywide because the evacuees relocated to various parts of the country.^1^ This study only included adults aged <75 years at the time of follow-up health check-ups to evaluate lifestyle habits. A total of 21,882 adults (9,689 men and 12,193 women) underwent follow-up examinations after the disaster (median follow-up duration, 1.4 years). Moreover, 2,209 participants with insufficient data on waist circumference before and after the disaster were excluded. Accordingly, 19,673 (8,654 men and 11,019 women) participants were eligible for this study. Informed consent was obtained from community representatives to conduct an epidemiologic study based on the guidelines of the Council for International Organizations of Medical Science.^16^ This study was approved by the Ethics Committee of Fukushima Medical University (#1916).

### Measures and definitions

Waist circumference was measured at the end of a normal expiration, with the measuring tape positioned midway between the lowest rib and the iliac crest or at the level of noticeable waist narrowing. Height was measured while the participants were on their feet wearing socks, and weight was obtained while the participants were wearing light clothing. Moreover, BMI was calculated as weight (kg) divided by height (m^2^). Abdominal obesity was defined as waist circumference of ≥85 cm for men and ≥90 cm for women, which is the standard Japanese definition.^17^ Overweight was defined as a BMI of ≥25 kg/m^2^. The specific medical check-ups included the standard questionnaire.^18^ Lifestyle-related data were obtained from the lifestyle-related items included in this questionnaire.

Cigarette smoking, alcohol intake, physical activity, adequate sleep, and diet were classified into two categories (yes or no). Moderate/heavy drinking was defined as consumption of >22 g of alcohol per day. Physical activity was assessed using the question ‘Are you performing walking or an equivalent exercise for 60 minutes or more in daily life?’ Sleeping was assessed using the question ‘Do you achieve enough relaxation by sleeping?’ Dietary habit was evaluated using the questions ‘Do you eat dinner 2 hours before bedtime three times or more per week?’, ‘Do you eat a snack after dinner for three times or more per week?’ and ‘Do you skip breakfast for three times or more per week?’ Eating speed was assessed using the question ‘Do you eat faster than others?’ and classified into three categories: fast, medium and slow.

### Statistical analysis

Student’s paired *t*-test was used to compare mean values, and the McNemar test was utilised to compare the proportion of individuals who had abdominal obesity.

Changes in data before and immediately after the disaster were compared using Student’s paired *t*-test and the Wilcoxon signed-rank test. The odds ratios (ORs) and 95% confidence intervals (CIs) were calculated using a logistic regression model. The potential confounding factors included age (<65 or ≥65 years), sex, baseline waist circumference, evacuation and lifestyle habits (current smoking, alcohol intake, daily physical activity, adequate sleep, eating habits before bedtime, eating snack after dinner, skipping breakfast, and eating speed [slow, normal, or fast]). To address the potential bias arising from missing data, multiple imputation was used to account for lifestyle-related missing data.^19^ All analyses were conducted using the Statistical Package for the Social Sciences software for Windows version 27.0 (IBM Corp., Armonk, NY, USA). All tests were two sided, and a *P*-value <0.05 was considered statistically significant.

## RESULTS

Changes in waist circumference, body weight, BMI, proportion of individuals with abdominal obesity and overweight and lifestyle habits before and after the disaster are shown in Table 1. After the disaster, the mean waist circumference, body weight, and BMI of all participants increased from 84.2 to 84.6 cm, 58.0 to 58.7 kg and 23.5 to 23.8 kg/m^2^, respectively (all *P* < 0.001). When stratified according to evacuation, the clinical factors increased from 84.6 to 85.6 cm, 58.8 to 60.1 kg and 23.7 to 24.3 kg/m^2^, respectively, among evacuees after the disaster (all *P* < 0.001). After the disaster, among non-evacuees, the mean waist circumference changed from 84.0 to 84.1 cm (*P* = 0.166), and the body weight and BMI increased from 57.6 to 58.0 kg (*P* < 0.001) and 23.4 to 23.6 kg/m^2^ (*P* < 0.001), respectively. The proportions of individuals with abdominal obesity and overweight before and after the disaster were 34.2% and 36.6% and 29.2% and 33.3%, respectively (all *P* < 0.001). These proportions increased from 37.0% to 42.1% and 31.9% to 39.6%, respectively, among evacuees after the disaster (all *P* < 0.001). The proportion of non-evacuees with abdominal obesity increased from 32.8% to 34.0%, and the proportions of overweight residents increased from 27.9% to 30.3% after the disaster (all *P* < 0.001).

**Table 1.** Clinical and lifestyle characteristics of participants before and after the Great East Japan Earthquake

	All participants			Non-evacuees			Evacuees		
		
	Before	After	*P-value*	Before	After	*P-value*	Before	After	*P-value*
Number	19,673			13,248			6,425		
Sex, male/female	8,654/11,019			5,806/7,442			2,848/3,577		
Age, years	62.5	64.0		62.7	64.2		61.9	63.6	
Evacuation, %	—	32.7		—	—		—	—	
Waist, cm	84.2 (8.8)	84.6 (9.1)	<0.001	84.0 (8.7)	84.1 (9.0)	0.166	84.6 (9.0)	85.6 (9.2)	<0.001
Abdominal obesity (≥85 cm in men, ≥90 cm in women), %	34.2	36.6	<0.001	32.8	34.0	<0.001	37.0	42.1	<0.001
Body weight, kg	58.0 (10.3)	58.7 (10.6)	<0.001	57.6 (10.2)	58.0 (10.4)	<0.001	58.8 (10.4)	60.1 (10.8)	<0.001
BMI, kg/m^2^	23.5 (3.3)	23.8 (3.4)	<0.001	23.4 (3.2)	23.6 (3.3)	<0.001	23.7 (3.3)	24.3 (3.4)	<0.001
Overweight (BMI of ≥25 kg/m^2^), %	29.2	33.3	<0.001	27.9	30.3	<0.001	31.9	39.6	<0.001
Current smoking, %	14.9	13.1	<0.001	14.2	12.3	<0.001	16.5	14.7	<0.001
Moderate/heavy alcohol intake,^a^ %	24.9	25.2	0.114	24.0	24.6	0.016	26.6	26.3	0.543
Daily physical activity^b^, %	36.2	34.8	<0.001	35.4	34.3	0.015	37.7	35.8	<0.01
Adequate sleep, %	75.4	71.4	<0.001	75.7	73.7	<0.001	74.9	66.5	<0.001
Eating habit before bedtime, %	22.1	21.0	<0.01	21.6	20.4	<0.01	23.0	22.3	0.248
Eating snack after dinner, %	10.0	9.2	0.001	9.3	9.1	0.367	11.3	9.4	<0.001
Breakfast skipping, %	5.7	5.4	0.114	5.2	5.0	0.205	6.6	6.3	0.341
Fast eating, %	24.3	24.4	0.584	23.8	23.8	0.898	25.4	25.7	0.452

BMI, body mass index.

Data were presented as means (standard deviation) for continuous variables and percentage for categorical variables.

^a^Moderate/heavy alcohol intake was defined as drinking >22 g of alcohol per day.

^b^Daily physical activity was defined as walking or similar exercise ≥60 min daily.

Regarding lifestyle, the proportion of current smokers decreased and that of individuals with adequate daily physical activity and sleep decreased (all *P* < 0.001). There was a decrease in the number of overall participants, especially non-evacuees, who had a habit of eating before bedtime (*P* < 0.01). Among overall participants, especially evacuees, the proportion of individuals who had a snack after dinner decreased (*P* < 0.001).

Table 2 shows the multivariable-adjusted ORs and 95% CIs of lifestyle factors associated with the incidence of abdominal obesity after the disaster. A total of 1,518 individuals had abdominal obesity. Moreover, 849 evacuees and 669 non-evacuees presented with abdominal obesity. The prevalence of abdominal obesity after the disaster was positively associated with current smoking (OR 1.51; 95% CI, 1.28–1.79) and moderate/heavy drinking (OR 1.17; 95% CI, 1.01–1.37) in all participants. The positive association between the prevalence of abdominal obesity and current smoking were found in both evacuees and non-evacuees and in both men and women (*P* values for interaction all >0.05) (eTable 1).

**Table 2.** Multivariable-adjusted odds ratios and 95% CIs of abdominal obesity after the disaster for variables among participants without abdominal obesity before the disaster

	All participants	Non-evacuees	Evacuees
Number	12,944	8,898	4,046
Evacuees vs non-evacuees	2.24 (1.97–2.54)	—	—
Age, ≥65 years	1.07 (0.94–1.22)	1.10 (0.93–1.29)	1.02 (0.83–1.25)
Sex, male	1.41 (1.22–1.64)	1.10 (0.91–1.33)	2.05 (1.63–2.58)
Current smoking	1.51 (1.28–1.79)	1.59 (1.28–1.99)	1.40 (1.07–1.83)
Moderate/heavy alcohol intake	1.17 (1.01–1.37)	1.33 (1.08–1.63)	0.99 (0.77–1.26)
Daily physical activity	1.12 (0.99–1.27)	1.19 (1.01–1.41)	1.02 (0.83–1.25)
Adequate sleep	1.02 (0.88–1.18)	1.00 (0.83–1.21)	1.07 (0.85–1.35)
Eating habit before bedtime	1.11 (0.96–1.29)	1.10 (0.90–1.34)	1.12 (0.89–1.41)
Eating snack after dinner	1.18 (0.96–1.44)	0.98 (0.74–1.29)	1.48 (1.09–2.00)
Breakfast skipping	1.02 (0.79–1.33)	1.02 (0.72–1.46)	1.06 (0.72–1.55)
Fast eating	1.07 (0.93–1.23)	1.02 (0.85–1.23)	1.13 (0.90–1.41)

CI, confidence interval.

The *P*-value for the association between evacuation and smoking, alcohol intake, daily physical activity, sleep habit, eating habit before bed time, eating snack after dinner, breakfast skipping and eating speed in individuals with abdominal obesity were 0.618, 0.827, 0.451, 0.697, 0.549, 0.059, 0.860 and 0.482, respectively.

Table 3 shows the multivariable-adjusted ORs and 95% CIs of changes in lifestyle habits associated with the prevalence of abdominal obesity after the disaster. Participants who reported smoking cessation (OR 4.08; 95% CI, 3.00–5.55) and alcohol drinking (OR 1.21; 95% CI, 1.02–1.44) before and after the disaster and stopping snacking after dinner (OR 1.33; 95% CI, 1.03–1.72) and non-breakfast skipping (OR 1.48; 95% CI, 1.02–2.14) after the disaster were positively associated with abdominal obesity. The associations between smoking cessation and abdominal obesity were observed in both evacuees and non-evacuees and in both men and women (*P* values for interaction all >0.05) (eTable 2). The positive association between starting to smoke and abdominal obesity was observed only in women. Moreover, there were correlations between alcohol drinking and abdominal obesity among non-evacuees and men. Besides, there were correlations between stopping snacking after dinner and abdominal obesity among evacuees.

**Table 3.** Multivariable-adjusted odds ratios and 95% CIs of abdominal obesity after the disaster for changes in lifestyle factors among participants without abdominal obesity before the disaster stratified according to evacuation

	Before	After	Proportion (%)	All participants (*n* = 12,944)	Non-evacuees (*n* = 8,898)	Evacuees (*n* = 4,046)
Current smoking	No	No	85.7	Ref		
	No	Yes	0.7	1.38 (0.72–2.66)	1.73 (0.75–3.95)	0.91 (0.31–2.70)
	Yes	No	2.4	4.08 (3.00–5.55)	4.69 (3.14–6.99)	3.38 (2.06–5.54)
	Yes	Yes	11.2	1.16 (0.96–1.41)	1.21 (0.94–1.55)	1.07 (0.79–1.45)
Moderate/heavy alcohol intake	No	No	76.0	Ref		
	No	Yes	4.2	1.03 (0.71–1.48)	0.88 (0.49–1.59)	1.30 (0.74–2.28)
	Yes	No	3.4	1.19 (0.86–1.65)	1.51 (0.99–2.29)	0.89 (0.55–1.46)
	Yes	Yes	16.4	1.21 (1.02–1.44)	1.33 (1.06–1.66)	1.08 (0.82–1.41)
Daily physical activity	Yes	Yes	20.9	Ref		
	Yes	No	15.3	1.05 (0.84–1.30)	1.03 (0.70–1.52)	1.03 (0.72–1.48)
	No	Yes	13.9	0.95 (0.75–1.19)	0.87 (0.62–1.24)	1.05 (0.75–1.47)
	No	No	49.9	0.90 (0.76–1.06)	0.85 (0.68–1.08)	0.96 (0.72–1.27)
Adequate sleep	Yes	Yes	58.8	Ref		
	Yes	No	14.9	0.87 (0.70–1.08)	0.91 (0.68–1.21)	0.84 (0.60–1.18)
	No	Yes	11.2	0.98 (0.76–1.27)	0.93 (0.68–1.26)	1.06 (0.73–1.56)
	No	No	15.1	0.94 (0.75–1.16)	1.06 (0.81–1.39)	0.77 (0.57–1.05)
Eating habit before bedtime	No	No	70.1	Ref		
	No	Yes	10.1	0.98 (0.77–1.26)	1.01 (0.74–1.38)	1.00 (0.70–1.42)
	Yes	No	10.7	1.13 (0.90–1.41)	1.08 (0.81–1.44)	1.19 (0.85–1.66)
	Yes	Yes	9.1	1.10 (0.86–1.40)	1.09 (0.80–1.48)	1.07 (0.71–1.61)
Eating snack after dinner	No	No	85.3	Ref		
	No	Yes	5.0	1.31 (0.97–1.76)	1.28 (0.82–1.99)	1.35 (0.85–2.14)
	Yes	No	5.8	1.33 (1.03–1.72)	1.19 (0.81–1.74)	1.51 (1.02–2.25)
	Yes	Yes	3.9	1.03 (0.73–1.46)	0.73 (0.41–1.31)	1.64 (0.97–2.77)
Breakfast skipping	No	No	92.2	Ref		
	No	Yes	2.3	1.29 (0.86–1.93)	1.21 (0.66–2.20)	1.42 (0.78–2.61)
	Yes	No	2.7	1.48 (1.02–2.14)	1.47 (0.84–2.54)	1.50 (0.90–2.51)
	Yes	Yes	2.8	0.78 (0.52–1.18)	0.82 (0.48–1.41)	0.80 (0.42–1.51)
Fast eating	No	No	72.4	Ref		
	No	Yes	6.8	1.06 (0.82–1.37)	1.25 (0.89–1.76)	0.81 (0.53–1.23)
	Yes	No	5.9	1.07 (0.79–1.46)	0.98 (0.66–1.44)	1.20 (0.78–1.86)
	Yes	Yes	14.9	1.09 (0.91–1.31)	1.08 (0.86–1.35)	1.07 (0.81–1.42)

CI, confidence interval; *n*, number.

## DISCUSSION

This study first examined the association between lifestyle habits and incidence of abdominal obesity among residents in the evacuation zone of Fukushima after the Great East Japan Earthquake. Waist circumference, body weight, and BMI of the participants increased after the earthquake.

Previous studies on individuals who evacuated after major disasters have mainly reported changes in blood pressure and glucose levels.^20^^,^^21^ Moreover, in 2016, Ohira et al reported that the residents in the evacuation zone gained weight after the Great East Japan Earthquake.^2^ In contrast, studies on the occurrence of abdominal obesity are limited. The risk of hypertension, diabetes, dyslipidaemia, and cardiovascular disease is high among individuals with abdominal obesity.^6^ This study revealed that the proportion of people with abdominal obesity significantly increased after the earthquake. Particularly, the prevalence of obesity was high among evacuees. Therefore, changes in lifestyle habits associated with modifications in living circumstances probably had some impact.

Regarding the association between lifestyle changes before and after the disaster and abdominal obesity, smoking cessation was strongly associated with the increase in abdominal obesity. The decrease in smoking rate before and after the earthquake is greater compared to the national average,^22^ and it is highly possible that the earthquake triggered smoking cessation. Even in previous earthquakes, the frequency of smoking cessation after the disaster was high.^23^ The reasons for smoking cessation are not only related to one’s own health but also the earthquake itself, lack of place to smoke and the worries of family and friends.^23^ Nakano et al explored the smoking rate of victims of the Great East Japan Earthquake and found that elderly individuals and women tended to quit smoking.^14^ Smoking cessation should reduce the risk of developing lifestyle-related diseases and metabolic syndromes as it is a risk factor for such conditions, but it is consistent with previous reports that smoking cessation exacerbates abdominal obesity.^24^^,^^25^ It is reasonable to believe that abdominal obesity was caused by smoking cessation following the earthquake within the observation period of this study because it has been reported that an increase in waist circumference was observed within 1 year after the start of smoking cessation. Although smoking cessation reduces the risk of lifestyle-related diseases, the adverse effects of increasing waist circumference may eliminate the beneficial effect of smoking cessation.^24^^,^^25^ Hence, it is necessary to carefully monitor the progress of those who stopped smoking after the earthquake. This association was consistent with the observation made in previous studies showing that smoking cessation is an early risk factor for abdominal obesity.^26^

Moreover, alcohol drinking before and after the disaster was associated with the increase in abdominal obesity. Alcohol consumption was directly associated with total energy intake.^9^ A correlation between alcohol consumption and abdominal obesity was also observed, which is consistent with previous studies.^9^

Alcohol drinking was associated with abdominal obesity in men but not in women (eTable 2). This finding was attributed to the fact that there was only a small proportion of women with these habits and the variance was large.

The mechanism explaining the association between quitting snacks after dinner and increased abdominal obesity is unknown. However, the timing of taking snacks has changed due to unemployment correlated with the earthquake.^27^ Moreover, an association was observed between non-breakfast skipping after the disaster and abdominal obesity. Participants might have started eating breakfast for health benefits. However, the causal relationships should be investigated. Moreover, the content of their breakfast must be evaluated.

The present study has some limitations. First, there were missing data on items correlated with lifestyle habits, and 29.8% of the participants in this analysis had missing data on any of the lifestyle-related items. To address the possibility of selection bias from missing data, multiple imputation was used to account for lifestyle-related missing data. Second, the data used in this study lacked details of social factors, and these factors were not fully considered in this study. However, to account for the influence of social factors to the best possible extent, we conducted an analysis including the major social factor ‘evacuation’ in the covariates. Third, the lifestyle factors were based on a self-reported questionnaire and may not have been accurate. There is also a need to verify the reproducibility and validity of lifestyle-related items. Finally, because this study evaluated the changes for up to 2 years after the earthquake, the impact of lifestyle changes on physical indicators might have been underestimated. Moreover, although the effect of the earthquake on lifestyle can be specifically observed using the data immediately before and after the earthquake, some lifestyle-related items may require long-term observation to verify their relationship with abdominal obesity.

### Conclusion

The prevalence of abdominal obesity increased among the residents in the area affected by nuclear disaster. This result might have been affected by not only lifestyle habits before the disaster but also changes in these habits after the disaster. To prevent abdominal obesity and reduce its prevalence among these residents, more targeted and detailed research is required.

## SUPPLEMENTARY MATERIAL

eTable 1. Multivariable-adjusted odds ratios and 95% CIs of abdominal obesity after the disaster for variables among participants without abdominal obesity before the disaster stratified according to sex

eTable 2. Multivariable-adjusted odds ratios and 95% CIs of overweight and abdominal obesity after the disaster for changes in lifestyle factors among participants without abdominal obesity before the disaster stratified according to sex
