# Supplementary material for: Association Between Lifestyle Habits and the Prevalence of Abdominal Obesity After the Great East Japan Earthquake: The Fukushima Health Management Survey
Source: J Epidemiol. 2022 Nov 5;32(11):496–501. doi: 10.2188/jea.JE20200597 (PMC9551296; doi:10.2188/jea.JE20200597)
Supplement: Supplementary file 1 [file je-32-496-s001.pdf]

eTable 1. Multivariable-adjusted odds ratios and 95% CIs of abdominal obesity after the disaster for variables among participants without abdominal obesity before the disaster stratified according to sex

	Men	Women
Number	4,412	8,532
Evacuees vs. non-evacuees	2.76 (2.31–3.29)	1.80 (1.49–2.16)
Age, ≥65 years	1.01 (0.85–1.21)	1.13 (0.94–1.36)
Current smoking, yes	1.50 (1.25–1.82)	1.74 (1.15–2.62)
Moderate/heavy alcohol intake, yes	1.19 (1.00–1.42)	1.06 (0.73–1.55)
Daily physical activity, yes	1.12 (0.94–1.34)	1.11 (0.92–1.34)
Adequate sleep, yes	1.16 (0.94–1.39)	0.92 (0.75–1.13)
Eating habit before bedtime, yes	1.14 (0.94–1.39)	1.08 (0.85–1.36)
Eating snacks after dinner, yes	1.21 (0.89–1.65)	1.15 (0.87–1.51)
Breakfast skipping, yes	0.90 (0.63–1.27)	1.22 (0.82–1.80)
Fast eating, yes	1.00 (0.82–1.22)	1.14 (0.92–1.39)

CI, confidence interval.

The *p*-value for the association between sex and smoking, alcohol intake, daily physical activity, sleep habit, eating habit before bed time, eating snacks after dinner, breakfast skipping and eating speed in individuals with abdominal obesity were 0.360, 0.795, 0.610, 0.087, 0.721, 0.988, 0.223 and 0.348, respectively.

eTable 2. Multivariable-adjusted odds ratios and 95% CIs of overweight and abdominal obesity after the disaster for changes in lifestyle factors among participants without abdominal obesity before the disaster stratified according to sex

			Men		Women	
	Before	After	Proportion (%)	All participants (n=4,412)	Proportion (%)	All participants (n=8,532)
Current smoking	No	No	67.6	Ref	95.1	Ref
	No	Yes	1.2	0.89 (0.39–2.00)	0.4	3.77 (1.25–11.36)
	Yes	No	5.4	4.38 (3.10–6.18)	0.9	3.09 (1.40–6.80)
	Yes	Yes	25.8	1.12 (0.91–1.38)	3.6	1.52 (0.94–2.45)
Moderate/heavy alcohol intake	No	No	46.7	Ref	91.1	Ref
	No	Yes	5.4	0.88 (0.54–1.42)	3.6	1.27 (0.77–2.11)
	Yes	No	6.3	1.25 (0.83–1.88)	1.9	0.95 (0.51–1.79)
	Yes	Yes	41.6	1.21 (1.00–1.47)	3.4	1.16 (0.72–1.89)
Daily physical activity	Yes	Yes	25.3	Ref	18.6	Ref
	Yes	No	15.2	1.15 (0.80–1.63)	15.4	0.94 (0.68–1.29)
	No	Yes	14.6	1.03 (0.78–1.37)	13.5	0.87 (0.60–1.25)
	No	No	44.9	0.90 (0.70–1.15)	52.5	0.89 (0.69–1.16)
Adequate sleep	Yes	Yes	63.0	Ref	56.6	Ref
	Yes	No	14.2	0.86 (0.63–1.16)	15.3	0.89 (0.67–1.18)
	No	Yes	10.5	0.90 (0.66–1.24)	11.6	1.06 (0.73–1.52)
	No	No	12.3	0.77 (0.57–1.04)	16.5	1.07 (0.80–1.43)
Eating habit before bedtime	No	No	62.6	Ref	74.0	Ref
	No	Yes	12.3	0.87 (0.63–1.21)	9.0	1.15 (0.75–1.76)
	Yes	No	12.0	1.10 (0.82–1.48)	10.0	1.16 (0.85–1.59)
	Yes	Yes	13.0	1.14 (0.86–1.51)	7.1	1.04 (0.69–1.56)
Eating snack after dinner	No	No	87.2	Ref	84.3	Ref

	No	Yes	4.7	1.27 (0.85–1.92)	5.2	1.34 (0.84–2.13)
	Yes	No	4.9	1.44 (0.98–2.12)	6.3	1.26 (0.89–1.79)
	Yes	Yes	3.1	1.00 (0.59–1.68)	4.2	1.07 (0.68–1.68)
Breakfast skipping	No	No	91.1	Ref	92.8	Ref
	No	Yes	2.5	1.63 (0.97–2.72)	2.2	1.00 (0.51–1.96)
	Yes	No	2.8	1.47 (0.88–2.43)	2.6	1.50 (0.89–2.54)
	Yes	Yes	3.6	0.71 (0.42–1.19)	2.4	0.95 (0.50–1.80)
Fast eating	No	No	70.9	Ref	73.2	Ref
	No	Yes	7.9	1.09 (0.76–1.56)	6.3	1.00 (0.68–1.47)
	Yes	No	6.1	0.84 (0.55–1.27)	5.8	1.36 (0.89–2.09)
	Yes	Yes	15.1	1.10 (0.86–1.41)	14.8	1.07 (0.82–1.40)

CI, confidence interval; n, number.
